# Plasmodium falciparum Uses gC1qR/HABP1/p32 as a Receptor to Bind to Vascular Endothelium and for Platelet-Mediated Clumping

**DOI:** 10.1371/journal.ppat.0030130

**Published:** 2007-09-28

**Authors:** Anup Kumar Biswas, Abdul Hafiz, Bhaswati Banerjee, Kwang Sik Kim, Kasturi Datta, Chetan E Chitnis

**Affiliations:** 1 Malaria Group, International Centre for Genetic Engineering and Biotechnology, New Delhi, India; 2 School of Environmental Sciences, Jawaharlal Nehru University, New Delhi, India; 3 Department of Pediatrics, Johns Hopkins University School of Medicine, Baltimore, Maryland, United States of America; Center for Global Health and Diseases, United States of America

## Abstract

The ability of Plasmodium falciparum–infected red blood cells (IRBCs) to bind to vascular endothelium, thus enabling sequestration in vital host organs, is an important pathogenic mechanism in malaria. Adhesion of P. falciparum IRBCs to platelets, which results in the formation of IRBC clumps, is another cytoadherence phenomenon that is associated with severe disease. Here, we have used in vitro cytoadherence assays to demonstrate, to our knowledge for the first time, that P. falciparum IRBCs use the 32-kDa human protein gC1qR/HABP1/p32 as a receptor to bind to human brain microvascular endothelial cells. In addition, we show that P. falciparum IRBCs can also bind to gC1qR/HABP1/p32 on platelets to form clumps. Our study has thus identified a novel host receptor that is used for both adhesion to vascular endothelium and platelet-mediated clumping. Given the association of adhesion to vascular endothelium and platelet-mediated clumping with severe disease, adhesion to gC1qR/HABP1/p32 by P. falciparum IRBCs may play an important role in malaria pathogenesis.

## Author Summary

Adhesion of Plasmodium falciparum–infected red blood cells (IRBCs) to the endothelium lining the capillaries of vital host organs can obstruct blood circulation and is an important pathogenic mechanism in malaria. Adhesion of P. falciparum IRBCs to platelets results in the formation of IRBC clumps that can also obstruct blood flow and is implicated in severe malaria. Here, we have identified a novel cytoadherence receptor that is found on both endothelial cells and platelets. We demonstrate, for the first time to our knowledge, that P. falciparum IRBCs use the 32-kDa human protein gC1qR/HABP1/p32 as a receptor to bind to human endothelial cells, including brain microvascular endothelial cells. In addition, we show that P. falciparum IRBCs can bind to gC1qR/HABP1/p32 on platelets to form clumps. Our study has thus identified a novel host receptor that is used for both adhesion to vascular endothelium and platelet-mediated clumping. Given the association of these cytoadherence phenomena with severe disease, our study opens the door to investigations on the role of adhesion of P. falciparum IRBCs to gC1qR/HABP1/p32 in malaria pathogenesis.

## Introduction

Malaria continues to be a major public health problem in many parts of the tropical world, with approximately 500 million malaria cases reported annually that result in 1–2 million deaths every year [1,2]. Deaths from malaria mainly occur in young children living in sub-Saharan Africa and are caused by infection with P. falciparum. One of the important virulence mechanisms associated with P. falciparum infection is the unique ability of P. falciparum trophozoites and schizonts to sequester in the vasculature of diverse host organs [3–7]. Sequestration of *P. falciparum–*infected red blood cells (IRBCs) in the microvasculature of the brain is associated with severe pathological outcome of cerebral malaria [3,5,7]. P. falciparum IRBCs can also bind to platelets to form platelet-mediated clumps, a cytoadherence phenomenon that is associated with severe disease [8–10].

Adhesion of IRBCs to vascular endothelium is mediated by interaction of the P. falciparum erythrocyte membrane protein-1 (PfEMP-1) family of variant surface antigens with host receptors [11–13]. The endothelial receptors used by P. falciparum for adhesion include thrombospondin (TSP) [14], CD36 [15], intercellular adhesion molecule-1 (ICAM-1) [16], platelet/endothelial cell adhesion molecule (PECAM/CD31) [17], vascular cell adhesion molecule-1 (VCAM-1) [18], endothelial leukocyte adhesion molecule-1 (ELAM-1) [18], normal immunoglobulin (IgG) [19], chondroitin sulfate A (CSA) [20,21], and hyaluronic acid (HA) [22]. Expression of ICAM-1 is upregulated on cerebrovascular endothelium [5,23], and P. falciparum IRBCs co-localize with ICAM-1 in cerebral vessels of patients who die of cerebral malaria [23], suggesting that adhesion to ICAM-1 plays a key role in cerebral sequestration. Adhesion of P. falciparum IRBCs to host vascular endothelium under flow conditions involves three distinct events, namely, margination, rolling, and static arrest/tethering, which may require multiple receptor–ligand interactions [24–26]. Adhesion to endothelial cells under flow requires binding of P. falciparum IRBCs to ICAM-1 as well as to CD36 [25]. Expression of ICAM-1 on brain endothelium is upregulated during blood stage P. falciparum infection [5,23]. However, the expression of CD36 on brain endothelial cells is minimal [23]. Platelets, which have been shown to accumulate in brain microvasculature of patients who die of cerebral malaria, express CD36 on their surface and may act as bridges for adhesion of P. falciparum IRBCs with brain vascular endothelium [27–29]. Alternatively, other as yet unidentified endothelial receptors may play a role in adhesion of P. falciparum IRBCs to cerebral capillaries. In case of platelet-mediated clumping, the only receptor identified for binding of IRBCs to platelets thus far is CD36 [9]. However, in previous studies, antibodies to CD36 could not completely disrupt clumps formed by some P. falciparum field isolates [9], suggesting that alternative host receptors may participate in platelet-mediated clumping.

Here, we report the identification of the 32-kDa human protein gC1qR/HABP1/p32 (referred to below as gC1qR/HABP1 for brevity) as a novel host receptor for cytoadherence by P. falciparum. gC1qR/HABP1 is a ubiquitously expressed membrane protein that was initially shown to bind to the globular “head” of complement component C1q [30] as well as to HA [31]. This receptor appears to bind to diverse ligands and has multiple functions [32,33]. It is expressed on diverse cell types, including endothelial cells [34], platelets [35], and dendritic cells [36], and is used as a cell surface receptor by microbial pathogens for pathogenic processes such as host cell entry [37,38] and suppression of immune function [39,40]. Given its localization on endothelial cells and platelets, we hypothesized that gC1qR/HABP1 may serve as a cytoadherence receptor for P. falciparum.

Here, we demonstrate that gC1qR/HABP1 is expressed on human brain microvascular endothelial cells (HBMECs) and can be used by P. falciparum as a receptor for cytoadherence. In addition, we show that P. falciparum IRBCs can bind gC1qR/HABP1 on platelets to form platelet-mediated IRBC clumps. Given the association of both of these cytoadherence phenotypes with severe malaria, this study identifies a novel host receptor that may play an important role in malaria pathogenesis.

## Results

### Binding of P. falciparum Laboratory Strains and Field Isolates to Endothelial Receptors gC1qR/HABP1, CD36, and ICAM-1

Recombinant human gC1qR/HABP1 was expressed in E. coli and purified to homogeneity (Figure S1). Recombinant gC1qR/HABP1 has the expected mobility on SDS-PAGE and has a purity of greater than 98%. Analysis by gel permeation chromatography reveals that the majority of gC1qR/HABP1 is trimeric, as predicted by the crystal structure ([41], Figure S1). Dimers and trimers of gC1qR/HABP1 purified by gel permeation chromatography migrate with the expected mobility for gC1qR/HABP1 monomers by SDS-PAGE (Figure S1). Recombinant gC1qR/HABP1 binds its known ligands, C1q (Figure S2) and HA (Figure S2), confirming that it is functional. P. falciparum laboratory strains as well as field isolates were tested for binding to recombinant gC1qR/HABP1 coated on plastic Petri plates (Table 1; Figure S3) and to CD36 and ICAM-1 expressed on the surface of stably transfected Chinese hamster ovary (CHO) cells (Table 1). Three of the eight P. falciparum field isolates tested bind gC1qR/HABP1 (Table 1). Of these, IGH-CR14 shows the most significant binding to gC1qR/HABP1 (Table 1) and was selected for further analysis. P. falciparum laboratory strain 3D7, which binds gC1qR/HABP1 (Table 1), was also used for further study. IGH-CR14 binds CD36 and ICAM-1 in addition to gC1qR/HABP1, whereas 3D7 binds CD36 and gC1qR/HABP1 but not ICAM-1 (Table 1). IGH-CR14 binds gC1qR/HABP1 monomers and trimers at similar levels (Figure S3). Soluble C1q blocks the binding of IGH-CR14 to gC1qR/HABP1, suggesting that binding sites on gC1qR/HABP1 used by IRBCs and C1q may be overlapping (Figure S4). HA has no effect on binding of IGH-CR14 to gC1qR/HABP1 (Figure S4).

**Table 1:**
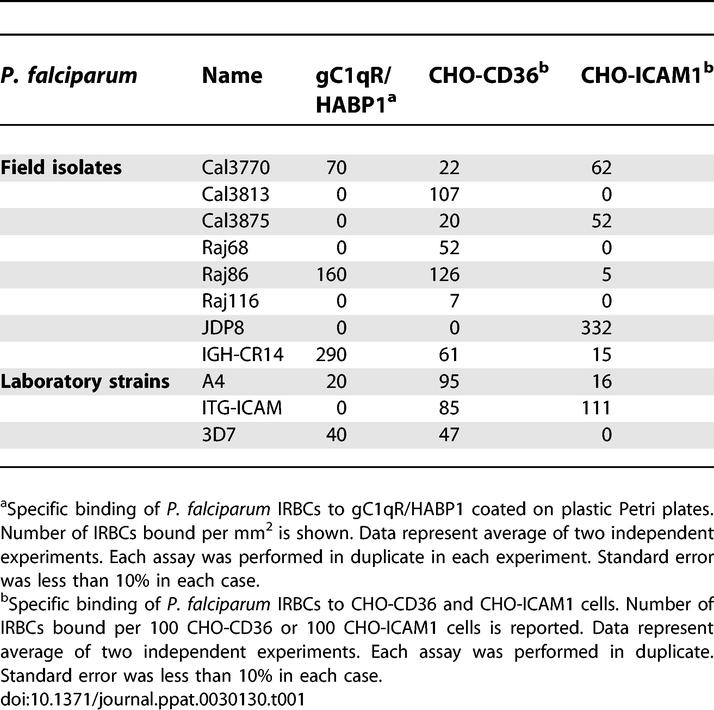
Adhesion of P. falciparum Field Isolates and Laboratory Strains to gC1qR/HABP1, CD36, and ICAM-1

Polymerase chain reaction–based analysis of two polymorphic antigens, MSP-1 and MSP-2, using methods described previously [42] confirmed that both IGH-CR14 and 3D7 contain single distinct genotypes (unpublished data). However, both IGH-CR14 and 3D7 may contain multiple variants with distinct binding phenotypes as a result of antigenic variation. In order to test if P. falciparum IRBCs, which bind gC1qR/HABP1, can also bind other receptors like CD36 or ICAM-1, we selected IGH-CR14 and 3D7 for binding to gC1qR/HABP1, separated binders (IGH-CR14+ and 3D7+) from non-binders (IGH-CR14− and 3D7−), and tested them in binding assays. As expected, IGH-CR14+ and 3D7+ show increased binding to gC1qR/HABP1, whereas IGH-CR14− and 3D7− display reduced binding to gC1qR/HABP1 compared to IGH-CR14 and 3D7, respectively (Table 2). The gC1qR/HABP1 binders, IGH-CR14+ and 3D7+, do not bind ICAM-1 or CD36, whereas IGH-CR14− retains binding to ICAM-1 and CD36, and 3D7− retains binding to CD36 (Table 2). These findings indicate that binding of *P. falciparum* IGH-CR14 and 3D7 to gC1qR/HABP1 is not linked to binding to ICAM-1 or CD36.

**Table 2:**
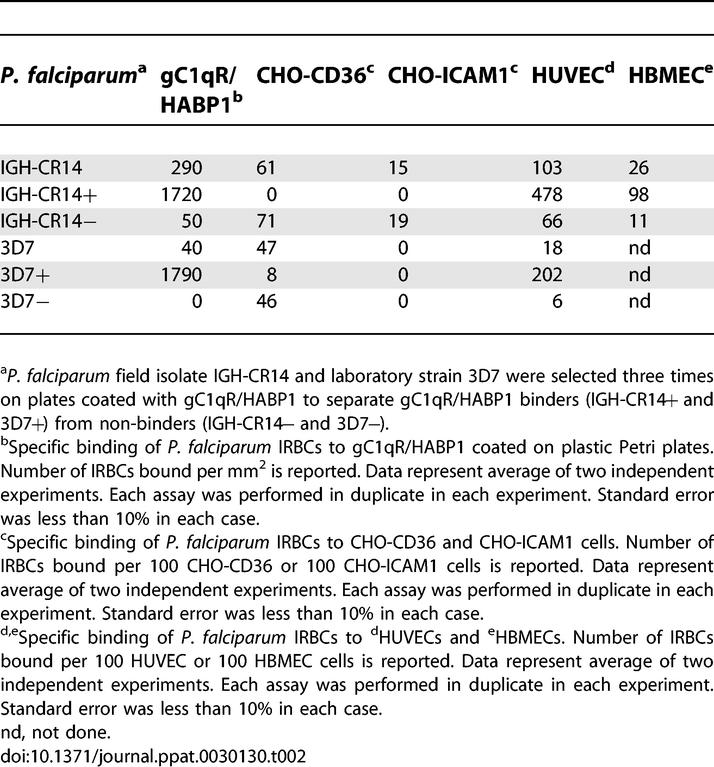
Adhesion of P. falciparum Isolates to gC1qR/HABP1, CD36, and ICAM-1, and Endothelial Cell Lines HUVEC and HBMEC

### Expression of gC1qR on Human Endothelial Cells

We have used mouse serum raised against recombinant gC1qR/HABP1 to detect gC1qR/HABP1 on the surface of human umbilical vein endothelial cells (HUVECs), immortalized HBMECs, and primary brain microvascular cells (PBMECs) by flow cytometry. Anti-gC1qR/HABP1 mouse serum recognizes a single band of the expected size (32 kDa) in whole cell lysates as well as in membrane preparations of HUVECs by western blotting (Figure S5). Moreover, anti-gC1qR/HABP1 mouse serum detects gC1qR/HABP1 on the surface of HUVECs, HBMECs, and PBMECs by flow cytometry (Table S1). Unlike ICAM-1, surface expression of gC1qR/HABP1 is not significantly upregulated on the surface of HUVECs, HBMECs, and PBMECs following treatment with TNF-α (Table S1). CD36 is not detected on the surface of HUVECs, HBMECs, and PBMECs before or after treatment with TNF-α (Table S1).

### P. falciparum IGH-CR14+ and 3D7+ Use gC1qR/HABP1 as a Receptor to Bind Endothelial Cells

In order to explore if P. falciparum IRBCs use gC1qR/HABP1 to bind endothelial cells, we tested IGH-CR14 and 3D7 for binding to HUVECs and HBMECs. We also tested whether selection of IGH-CR14 and 3D7 for binding to gC1qR/HABP1 results in enhanced binding to endothelial cells. IGH-CR14+ and 3D7+ show increased binding to both gC1qR/HABP1 and HUVECs compared to IGH-CR14 and 3D7 or the non-binders, IGH-CR14− and 3D7− (Table 2). The association of enhanced binding to gC1qR/HABP1 and HUVECs (Table 2) suggested that IGH-CR14+ and 3D7+ use gC1qR/HABP1 as a cell surface receptor to bind to HUVECs.

**Figure 1:**
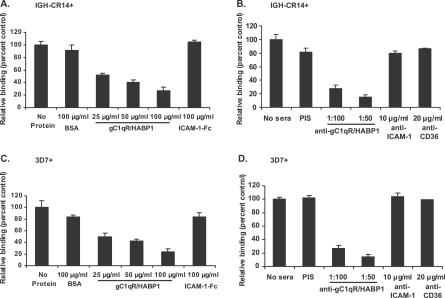
Inhibition of IRBCs Binding to HUVECs with Soluble gC1qR/HABP1 and Mouse Antiserum against gC1qR/HABP1 Binding of IRBCs to HUVECs in the presence of soluble proteins is expressed as relative binding compared to binding in absence of any protein (No protein). Binding in the presence of serum is expressed as relative binding compared to binding in absence of any serum (No sera). Recombinant gC1qR/HABP1 inhibits binding of IGH-CR14+ (A) and 3D7+ (C) to HUVECs in a dose-dependent manner. Recombinant ICAM-1-Fc and BSA have no effect on binding of IGH-CR14+ (A) and 3D7+ (C) to HUVECs. Anti-gC1qR/HABP1 mouse serum inhibits binding of IGH-CR14+ (B) and 3D7+ (D) to HUVECs. Pre-immune mouse serum (PIS), anti-ICAM-1 monoclonal antibody 15.2, and anti-CD36 monoclonal antibody SMΦ do not have any effect on binding of IGH-CR14+ (B) and 3D7+ (D) to HUVECs. The number of IRBCs bound per 100 HUVECs was scored in each assay. All data are averages (± standard error) derived from two independent experiments. Each assay was performed in duplicate. Binding of IGH-CR14+ and 3D7+ IRBCs in absence of any protein or serum was in the range of 150–200 IRBCs bound to 100 HUVECs.

In order to confirm that binding of IGH-CR14+ and 3D7+ to HUVECs was mediated by gC1qR/HABP1, we tested whether soluble gC1qR/HABP1, as well as anti-gC1qR/HABP1 mouse serum, can inhibit binding of IGH-CR14+ and 3D7+ to HUVECs. Soluble gC1qR/HABP1 blocks the binding of both IGH-CR14+ and 3D7+ to HUVECs in a dose-dependent manner, whereas bovine serum albumin (BSA) and recombinant ICAM1-Fc have no effect on binding (Figure 1). Anti-gC1qR/HABP1 mouse serum also blocks binding of both IGH-CR14+ and 3D7+ to HUVECs, whereas pre-immune mouse serum and antibodies directed against ICAM-1 or CD36 have no effect on binding (Figure 1). These findings demonstrated that binding of IGH-CR14+ and 3D7+ to HUVECs is mediated by gC1qR/HABP1.

**Figure 2:**
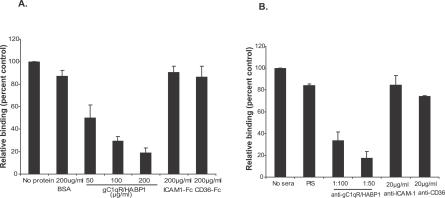
Inhibition of IRBCs Binding to HBMECs with Soluble gC1qR/HABP1 and Mouse Antiserum against gC1qR/HABP1 Binding of IRBCs to HBMECs in the presence of soluble proteins is expressed as relative binding compared to binding in absence of any protein (No protein). Binding in the presence of serum is expressed as relative binding compared to binding in absence of any serum (No serum). Recombinant gC1qR/HABP1 inhibits binding of IGH-CR14+ (A) to HBMECs in a dose-dependent manner. Recombinant ICAM-1-Fc, CD36-Fc, and BSA have no effect on binding of IGH-CR14+ (A). Anti-gC1qR/HABP1 mouse serum inhibits binding of IGH-CR14+ (B) to HBMECs. Pre-immune mouse serum (PIS), anti-ICAM-1 monoclonal antibody (clone 15.2), and anti-CD36 monoclonal antibody (clone SMΦ) do not have any effect on binding of IGH-CR14+ (B) to HBMECs. The number of IRBCs bound to 100 HBMEC cells was scored in each assay. All data are averages (± standard error) derived from two independent experiments. Each assay was performed in duplicate. Binding of IGH-CR14+ in absence of any protein or serum was in the range of 90–100 IRBCs bound to 100 HBMECs.

The gC1qR/HABP1 binder IGH-CR14+ also shows increased binding to HBMECs compared to IGH-CR14 and IGH-CR14− (Table 2). Binding of IGH-CR14+ to HBMECs is inhibited by soluble gC1qR/HABP1 but not by ICAM1-Fc or CD36-Fc (Figure 2). Binding of IGH-CR14+ to HBMECs is also inhibited by anti-gC1qR/HABP1 mouse serum but not by pre-immune mouse serum or monoclonal antibodies against ICAM-1 and CD36 (Figure 2). These findings demonstrate that IGH-CR14+ uses gC1qR/HABP1 as a receptor to bind to HBMECs.

### P. falciparum IGH-CR14+ and 3D7+ Use gC1qR/HABP1 as a Receptor for Platelet-Mediated Clumping of IRBCs

**Figure 3:**
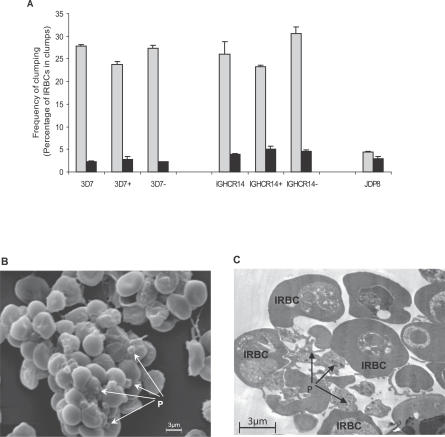
Platelet-Mediated Clumping of P. falciparum IRBCs (A) Frequency of platelet-mediated clumping in PRP and PPP. P. falciparum field isolate IGH-CR14, laboratory strain 3D7, and their derivatives, IGH-CR14+ and 3D7+, which bind gC1qR/HABP1, IGH-CR14−, and 3D7−, which bind CD36, and P. falciparum isolate JDP8, which does not bind gC1qR/HABP1 or CD36, were tested for clumping in the presence of PRP and PPP. Parasites in trophozoite and schizont stages were allowed to form clumps in the presence of PRP and PPP. Parasites were stained with acridine orange and the clumping frequency was determined by scoring the frequency of IRBCs found in clumps. Approximately 500 IRBCs were scored for each parasite. The frequency of clumping in the presence of PRP (grey bars) and PPP (black bars) is shown. (B) Scanning electron micrograph of platelet-mediated clumps formed by IGH-CR14+. Clumps formed by IGH-CR14+ IRBCs in the presence of PRP were analyzed by scanning electron microscopy. Electron micrograph shows several platelets (marked P), which bridge IRBCs in the clumps. (C) Transmission electron micrograph of platelet-mediated clumps formed by IGH-CR14+. Clumps formed by IGH-CR14+ IRBCs in the presence of PRP were analyzed by transmission electron microscopy. Only IRBCs are present in the clumps. The IRBCs are closely associated with platelets (P) in the clumps.

Mouse serum raised against gC1qR/HABP1 was used to detect expression of gC1qR/HABP1 on the surface of platelets by flow cytometry. P-selectin (CD62) was used as a marker for platelet activation. Whereas gC1qR/HABP1 is detected on the surface of both resting and activated platelets, P-selectin is only expressed on the surface of activated platelets (Table S2). Given the presence of gC1qR/HABP1 on the surface of platelets, we examined whether P. falciparum could use gC1qR/HABP1 as a receptor for platelet-mediated IRBC clumping. IGH-CR14, IGH-CR14+, and IGH-CR14− were tested for formation of clumps in the presence of platelet-rich plasma (PRP) and platelet-poor plasma (PPP). All three parasites form clumps in the presence of PRP, whereas no clumps are seen in the presence of PPP (Figure 3). Similarly, 3D7, 3D7+, and 3D7− form clumps in the presence of PRP (Figure 3). The P. falciparum isolate JDP8, which binds ICAM-1 and does not bind gC1qR/HABP1 or CD36, does not form clumps in PRP or PPP. IGH-CR14, IGH-CR14−, 3D7, and 3D7− bind CD36 (Table 2), which is a known receptor for platelet-mediated clumping. IGH-CR14+ and 3D7+ do not bind CD36, but bind gC1qR/HABP1 (Table 2). Analysis of clumps formed by IGH-CR14+ using scanning and transmission electron microscopy confirmed the presence of platelets in the clumps (Figure 3), suggesting that IGH-CR14+ IRBCs use gC1qR/HABP1 as a receptor to form platelet-mediated clumps.

**Figure 4:**
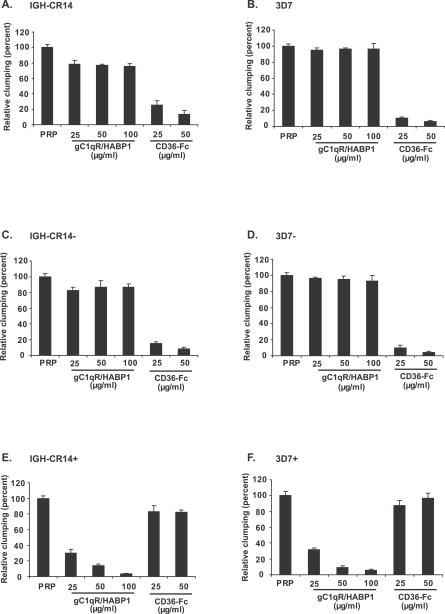
Inhibition of Platelet-Mediated Clumping of IRBCs by Soluble gC1qR/HABP1 and CD36-Fc Parasite cultures in trophozoite and schizont stages were allowed to form clumps in the presence of PRP. Parasites were stained with acridine orange and the clumping frequency was determined by scoring the frequency of IRBCs found in clumps. Approximately 2,000 to 3,000 IRBCs were scored. Parasite cultures were pre-incubated with recombinant gC1qR/HABP1 or CD36-Fc at different concentrations prior to use in clumping assays to test their ability to inhibit clumping. Clumping frequency in the presence of gC1qR/HABP1 or CD36-Fc is expressed as relative clumping compared to clumping in the presence of PRP alone. Data represent the average (± standard error) of two independent experiments. Each assay was performed in duplicate. (A) IGH-CR14, (B) 3D7, (C) IGH-CR14−, (D) 3D7−, (E) IGH-CR14+, (F) 3D7+.

**Figure 5:**
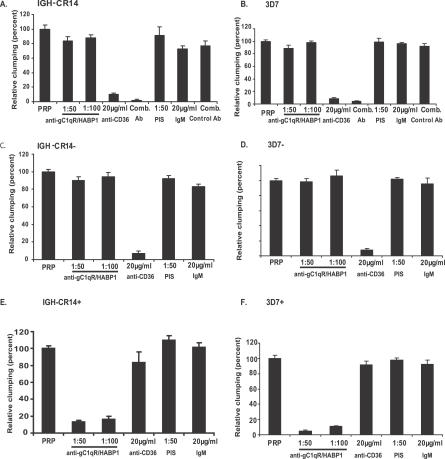
Inhibition of Platelet-Mediated Clumping of IRBCs by Mouse Serum Directed against gC1qR/HABP1 and Monoclonal Antibody against CD36 Parasite cultures in trophozoite and schizont stages were allowed to form clumps in the presence of PRP. Parasites were stained with acridine orange and the clumping frequency was determined by scoring the percentage of IRBCs found in clumps. Approximately 2,000 to 3,000 IRBCs were scored. The ability of anti-gC1qR/HABP1 mouse serum (anti-gC1qR/HABP1) or anti-CD36 mouse monoclonal IgM antibody (anti-CD36, clone SMΦ) to inhibit clump formation was tested by pre-incubating platelets with antibodies prior to use in clumping assays. Pre-immune serum (PIS) from mice immunized with gC1qR/HABP1 and purified mouse IgM were used as controls. Clumping frequency in the presence of anti-gC1qR/HABP1 or anti-CD36 is expressed relative to clumping in the presence of PRP alone. Data represent average (± standard error) of two independent experiments. Each assay was performed in duplicate. (A) IGH-CR14, (B) 3D7, (C) IGH-CR14−, (D) 3D7−, (E) IGH-CR14+, (F) 3D7+.

In order to confirm the identity of the receptor used by IGH-CR14+ and 3D7+ for platelet-mediated clumping we tested the ability of soluble gC1qR/HABP1 and CD36-Fc, as well as antibodies directed against gC1qR/HABP1 and CD36, to inhibit clumping. Both CD36-Fc and anti-CD36 monoclonal antibodies block the clumping of IGH-CR14, IGH-CR14−, 3D7, and 3D7− (Figures 4 and 5). Soluble gC1qR/HABP1 and anti-gC1qR/HABP1 mouse serum does not inhibit clumping of these parasites (Figures 4 and 5). These findings indicate that IGH-CR14, IGH-CR14−, 3D7, and 3D7− primarily use CD36 on platelets to form clumps. Conversely, soluble gC1qR/HABP1 and anti-gC1qR/HABP1 mouse serum block clumping of IGH-CR14+ and 3D7+ parasites, whereas CD36-Fc and anti-CD36 monoclonal antibodies do not have any inhibitory effect on clumping of IGH-CR14+ and 3D7+ parasites (Figures 4 and 5). These studies confirm that both IGH-CR14+ and 3D7+ use gC1qR/HABP1 as a receptor for platelet-mediated clumping.

## Discussion

Adhesion of P. falciparum IRBCs to endothelial receptors, which enables sequestration in host organs, and binding to platelets, which produces IRBC clumps, are important pathogenic mechanisms in malaria [4–10]. Here, we report the identification of the 32-kDa human protein gC1qR/HABP1 as a novel cytoadherence receptor for adhesion of P. falciparum IRBCs to both endothelial cells and platelets.

gC1qR/HABP1 is synthesized as a 282–amino acid pre-pro protein, which contains a 73–amino acid long N-terminal mitochondrial targeting sequence [43,44]. gC1qR/HABP1 is found in mitochondria and also on the surface of mammalian cells. There are other examples of proteins that have mitochondrial localization sequences and are found in other cellular locations in addition to mitochondria [45]. For example, mitochondrial aspartate aminotransferase (ApsAT) is found in the mitochondria as well as on the plasma membrane of adipocytes, where it is involved in binding and uptake of fatty acids [45]. Another mammalian protein, Slit3, whose homolog in *Drosophila* is involved in developmental regulation, is predominantly a mitochondrial protein having an N-terminal mitochondrial targeting sequence, but is also shown to be expressed on epithelial cell surfaces [46]. The mechanisms by which these proteins translocate to the cell surface and to the mitochondria are not known. Given the presence of the mitochondrial targeting sequence and absence of a transmembrane domain or consensus glcophosphatidyl inositol (GPI)–anchoring signature sequence, the surface localization of gC1qR/HABP1 is intriguing. The localization of gC1qR/HABP1 on the surface of diverse human cells has been demonstrated unequivocally both here and previously [31–34,47,48]. We have demonstrated here that mouse serum raised against recombinant gC1qR/HABP1 specifically reacts with a protein of the expected size for gC1qR/HABP1 (32 kDa) in whole cell lysates as well as in membrane fractions of HUVECs by western blotting (Figure S5). Anti-gC1qR/HABP1 mouse serum detects the presence of gC1qR/HABP1 on the surface of HUVECs, HBMECs, and PBMECs by flow cytometry (Table S1). The observation that gC1qR/HABP1 is expressed on the surface of microvascular endothelial cells suggests the possibility that it may be used as a receptor for cytoadherence by P. falciparum IRBCs. Given that the expression profile of the cytoadherence receptors gC1qR/HABP1, ICAM-1, and CD36 on HUVECs, HBMECs, and PBMECs is similar, we used HUVECs and HBMECs for adhesion assays with P. falciparum IRBCs.

We have demonstrated that P. falciparum laboratory strains as well as field isolates can bind to recombinant gC1qR/HABP1 (Table 1; Figure S3). Selection of P. falciparum IGH-CR14 and 3D7 for binding to gC1qR/HABP1 allowed separation of gC1qR/HABP1 binders IGH-CR14+ and 3D7+, and non-binders IGH-CR14− and 3D7−. Selection of IGH-CR14 and 3D7 for binding to gC1qR/HABP1 resulted in increased binding of IRBCs to HUVECs and HBMECs (Table 2), suggesting that these parasites use gC1qR/HABP1 to bind to human endothelial cells. Indeed, recombinant gC1qR/HABP1, as well as anti-gC1qR/HABP1 mouse serum, blocked the binding of IGH-CR14+ to HUVECs and HBMECs (Figures 1 and 2), confirming that these parasites use gC1qR/HABP1 as a receptor for adhesion of IRBCs to endothelial cells. The demonstration that P. falciparum IRBCs can use gC1qR/HABP1 as a receptor to bind to microvascular endothelial cells suggests that adhesion to gC1qR/HABP1 may play a role in parasite sequestration in vivo.

Another distinct cytoadherence phenotype that is associated with severe malaria is platelet-mediated clumping of IRBCs. CD36, which is expressed on both resting and activated platelets, has been identified as a receptor for platelet-mediated clumping [9]. However, in a previous study, clumps formed by some P. falciparum field isolates could not be disrupted completely by anti-CD36 antibodies [9], suggesting that other unidentified receptors on platelets might also mediate clumping of IRBCs. Previous studies have suggested that gC1qR/HABP1 is expressed on the surface of activated platelets [35]. Here, we have demonstrated that gC1qR/HABP1 is also expressed on resting platelets (Table S2). Expression of gC1qR/HABP1 on the surface increases upon activation of platelets (Table S2). IGH-CR14+ and 3D7+, which bind to gC1qR/HABP1 but not to CD36, formed clumps in the presence of platelets. Formation of clumps by IGH-CR14+ and 3D7+ was inhibited by soluble gC1qR/HABP1 and anti-gC1qR/HABP1 antibodies (Figures 4 and 5). These observations demonstrate that P. falciparum IRBCs can use gC1qR/HABP1 as an alternative receptor to bind to platelets and form clumps.

The parasite ligands that mediate adhesion of IRBCs to gC1qR/HABP1 remain to be identified. Previous studies have demonstrated that the PfEMP-1 family of variant surface antigens encoded by *var* genes mediates interactions with a diverse range of host receptors to enable adhesion to host endothelium and sequestration in host organs [4,6,12,13]. It is likely that PfEMP-1 may also mediate adhesion to gC1qR/HABP1. Identification of *var* genes that are differentially transcribed in gC1qR/HABP1 binding parasites may enable the identification of the PfEMP-1 variant that is responsible for adhesion to gC1qR/HABP1.

In summary, we have shown that P. falciparum IRBCs use gC1qR/HABP1 as a receptor to bind vascular endothelium and platelets. The observation that P. falciparum can use gC1qR/HABP1 as a receptor to bind HBMECs, a cell line derived from brain microvascular endothelial cells, raises the possibility that adhesion of IRBCs to this novel receptor may be important for sequestration in brain microvasculature and cerebral malaria. The contribution of IRBC adhesion to gC1qR/HABP1 to platelet-mediated clumping and severe disease also needs to be examined. Comparison of the cytoadherence phenotypes of P. falciparum isolates collected from patients with mild and severe malaria may allow us to test whether adhesion to gC1qR/HABP1 is associated with an increased risk of severe malaria.

## Materials and Methods

### Materials.

All chemicals used in the study were from Sigma (http://www.sigmaaldrich.com/) unless otherwise indicated.

### Parasites.

Indian P. falciparum field isolates were collected from *P. falciparum–*infected individuals in Calcutta (Cal3770, Cal3813, Cal3875), Rajasthan (Raj68, Raj86, Raj116), Jagdalpur, Madhya Pradesh (JDP8), and Rourkela, Orissa (IGH-CR14), and cryopreserved in the Malaria Parasite Bank at the National Institute of Malaria Research, Delhi, India. P. falciparum field isolates and laboratory strains (A4, ITG-ICAM, and 3D7) were cultured in RPMI 1640 (Invitrogen, http://www.invitrogen.com/) supplemented either with 10% heat-inactivated O+ pooled human sera or 5% Albumax I (Invitrogen) using O+ RBCs in an environment containing 5% O_2_, 5% CO_2_, and 90% N_2_. Cultures were synchronized by sorbitol treatment as previously described [49]. To select parasites for binding to gC1qR/HABP1, synchronized P. falciparum 3D7 and IGH-CR14 cultures in trophozoite/schizont stages were incubated for 1 h in bacteriological Petri plates coated with recombinant gC1qR/HABP1 as described below for adhesion assays. Unbound parasites were collected using a pipette and separated from bound parasites. Both bound and unbound parasites were cultured and subjected to selection for binding to gC1qR/HABP1 two more times to separate binders (3D7+ and IGH-CR14+) and non-binders (3D7− and IGH-CR14−).

### Cell lines.

Glycosaminoglycan biosynthesis–defective mutant Chinese hamster ovary cells (CHO-745) stably transfected to express human CD36 (CHO-CD36) and ICAM-1 (CHO-ICAM-1) on their surface [50] were kindly provided by Artur Scherf, Institut Pasteur, Paris, France. CHO cells were cultured in RPMI 1640 with 10% heat-inactivated fetal bovine serum (FBS). HUVECs were cultured in EBM2 bullet kit media (Cambrex Biosciences, http://www.cambrex.com/) on gelatin-coated flasks according to instructions provided by the supplier. Immortalized HBMECs were cultured as previously described [51]. PBMECs were cultured in EGM-2 media provided by the supplier (ScienCell Research Laboratories, http://www.sciencellonline.com/).

### Purification and characterization of recombinant human gC1qR/HABP1.

A DNA fragment encoding mature human gC1qR/HABP1 (amino acids 74–282) was cloned in pET30b vector (Invitrogen) using the NdeI and BamHI restriction enzyme cloning sites. Recombinant gC1qR/HABP1 was expressed in E. coli BL21(DE3) by induction with isopropyl-1-thio-β-galactosidase (IPTG) and purified from supernatants of lysed cells by ammonium sulfate fractionation followed by ion-exchange chromatography using UnoQ (Bio-Rad, http://www.bio-rad.com/) as described previously [39]. Binding of recombinant gC1qR/HABP1 to its ligands, C1q and HA, was tested in solid phase binding assays as follows. gC1qR/HABP1 and HA were biotinylated with sulfo-NHS-LC-biotin and biotin-LC-hydrazide, respectively, as described by the manufacturer (Pierce Biotechnology, http://www.piercenet.com/). Ninety-six-well plates were coated at 4 °C overnight with human C1q (250 ng per well). After blocking with 2% non-fat milk, the wells were incubated with varying concentrations of biotinylated gC1qR/HABP1. Bound biotin-gC1qR/HABP1 was detected with streptavidin-horse radish peroxidase (HRPO) using *o*-phenylene diamine dihydrochloride (OPD) as substrate. In order to test the binding of gC1qR/HABP1 to HA, ELISA plate wells were coated with recombinant gC1qR/HABP1 (250 ng per well) and incubated with different concentrations of biotin-HA. Bound biotin-HA was detected using streptavidin-HRPO and OPD.

### Adhesion assays with soluble proteins and stable CHO cell transfectants.

Ten microliters of purified gC1qR/HABP1 (100 μg/ml) was spotted on bacteriological Petri plates (Becton Dickinson, http://www.bd.com/), allowed to adsorb overnight at 4 °C in a humidified chamber and used for binding assays with parasite cultures as previously described for binding to soluble CD36 and ICAM-1 [52]. BSA was spotted as control. Trophozoite-schizont stage parasite cultures at ∼1% hematocrit and ∼5% parasitemia were incubated with gC1qR/HABP1-coated Petri plates to allow binding. Bound cells were fixed with 2% glutaraldehyde, stained with 5% Giemsa stain, and scored using a Nikon TE200 microscope with a 100× objective. The total number of IRBCs and uninfected RBCs (URBCs) were counted from seven randomly selected distinct fields in duplicate spots from two independent experiments. The number of URBCs bound to gC1qR/HABP1 and the number of IRBCs bound to BSA spots was subtracted from the number of IRBCs bound to gC1qR/HABP1 to get the number of specific binding events. Fewer than five URBCs bound gC1qR/HABP1 per mm^2^, and fewer than five IRBCs bound BSA per mm^2^.

CHO-745, CHO-CD36, and CHO-ICAM1 cells were grown in spots in tissue culture plates and tested for binding to P. falciparum trophozoite-schizont stage cultures using methods described earlier [52]. Bound IRBCs were fixed with 2% glutaraldehyde and detected by Giemsa staining. The number of IRBCs and URBCs bound to ∼200 CHO cells was scored in duplicate spots in two independent experiments. The number of URBCs bound to CHO-CD36 or CHO-ICAM1 was subtracted from bound IRBCs in each case. The number of IRBCs bound to CHO-745 was further subtracted from the number of IRBCs bound to CHO-CD36 and CHO-ICAM1 to obtain the number of specific binding events. Fewer than three URBCs bound to 100 CHO-CD36 or CHO-ICAM1 cells, and fewer than two IRBCs bound to 100 CHO-745 cells in all experiments. Specific binding of ten IRBCs or more per 100 CHO-ICAM1 or CHO-CD36 cell was therefore considered significant.

### Flow cytometry.

Flow cytometry was used to study the expression of gC1qR/HABP1, ICAM-1, and CD36 on the surface of HUVECs, HBMECs, and PBMECs before and after treatment with TNF-α (eBioscience, http://www.ebioscience.com/) and on the surface of resting and activated platelets.

HUVECs, HBMECs, and PBMECs were cultured as described above and were treated with TNF-α (15 ng/ml) for 24 h before analysis. Mouse serum raised against gC1qR/HABP1 (diluted 1:100), anti-ICAM-1 monoclonal antibody 15.2 (1 μg per 10^6^ cells; Serotec, http://www.serotec.co.uk/), and anti-CD36 monoclonal antibody SMΦ (1 μg per 10^6^ cells, Serotec) were used for detection of receptors by flow cytometry using the BD FACSCalibur System (Becton Dickinson).

Resting platelets were isolated from whole blood collected in citrate-phosphate-dextrose (CPD) as follows. PRP was separated by centrifugation of whole blood at 300*g* for 5 min at room temperature (RT). PRP was incubated in equal volume of CCAT buffer (7.7 mM citric acid, 95 mM trisodium citrate, 150 mM glucose, 5 mM adenosine, 3 mM theophylline) for 10 min at RT. Platelets were collected by centrifugation of PRP at 1,000*g* for 10 min at RT, washed once in CCAT buffer and resuspended in modified Hank's balanced salt buffer (136 mM NaCl, 5.3 mM KCl, 0.4 mM MgSO_4_.7H_2_O, 0.3 mM NaH_2_PO_4_.2H_2_O, 0.77 mM Na_2_HPO_4_, 0.44 mM KH_2_PO_4_, 0.5 mM MgCl_2_.6H_2_O, 5.5 mM glucose, 0.4 mM NaHCO_3_), and stored at RT until use. Resting platelets resuspended in RPMI 1640 were activated by treatment with thrombin (100 units/ml; Sigma Chemicals, http://www.sigmaaldrich.com/) for 30 min at 37 °C. Resting and activated platelets were fixed with 2% p-formaldehyde and 0.2% glutaraldehyde in phosphate buffered saline for 30 min at 4 °C. Mouse antiserum raised against gC1qR/HABP1 (diluted 1:100) and anti-P-selectin monoclonal IgG antibody CTB201 (1 μg per 10^6^ platelets; Santa Cruz Biotechnology, http://www.scbt.com/) were used for detection of receptors by flow cytometry using the BD FACSCalibur System (Becton Dickinson).

### Binding assay with endothelial cells and inhibition with soluble proteins and sera.

HUVECs and HBMECs were grown on gelatin-coated plates and used for binding assays with IRBCs following the same procedure used in case of CHO-CD36 and CHO-ICAM1 cells described above. For inhibition assays, either parasite cultures were pre-incubated with ICAM1-Fc (R&D Systems, http://www.rndsystems.com/), gC1qR/HABP1, and BSA, or HUVECs were pre-incubated with anti-gC1qR/HABP1 mouse serum or monoclonal antibodies directed against CD36 (clone SMΦ, Serotec) and ICAM-1 (clone 15.2, Serotec). Binding in the presence of proteins or serum was expressed as percent of binding in absence of any protein or serum.

### Platelet-mediated clumping assay and inhibition of clumping with soluble proteins and sera.

Platelet-mediated clumping assays were performed in the presence of PRP and PPP according to the method described previously [9]. IRBCs were labeled with acridine orange and the percentage of IRBCs present in clumps was determined by scoring ∼3,000 IRBCs at 20× magnification using a fluorescence microscope to determine frequency of clumping. A clump consists of three or more IRBCs as described previously [9]. All the clumps observed had fewer than 50 IRBCs. Parasite cultures were pre-incubated with soluble gC1qR/HABP1 or CD36-Fc (R&D Systems) for 10 min prior to adding PRP to test their ability to inhibit clumping. Antibodies directed against host proteins were added to PRP prior to incubation with parasite cultures to test their ability to block clumping.

### Electron microscopy.

Cells were fixed in 2.5% glutaraldehyde in 0.1 M phosphate buffer (pH 7.2) and processed according to methods described previously [9]. Samples were analyzed on a Morgagni 268D transmission electron microscope (FEI Philips, http://www.fei.com/) and LEO 435 VP scanning electron microscope (Leo Electron Microscopy, http://www.smt.zeiss.com/nts).

## Supporting Information

Figure S1 Characterization of Recombinant Human gC1qR/HABP1 (A) Purity of recombinant gC1qR/HABP1. Purified gC1qR/HABP1 was analyzed by SDS-PAGE under reducing conditions and detected by Coomassie staining. Different amounts of BSA (0.1 μg, 0.2 μg, 0.5 μg, and 1.0 μg) were used as control. Molecular weight markers are shown in kDa. (B) Recombinant gC1qR/HABP1 forms trimers. Recombinant purified gC1qR/HABP1 was analyzed by gel permeation chromatography using Superdex 200 HR10/30 column. Theoretical molecular mass of gC1qR/HABP1 is 23 kDa. Recombinant gC1qR/HABP1 primarily migrates as a trimer with molecular weight of ∼69 kDa. Blue dextran 2000 (2,000 kDa), BSA (67 kDa), ovalbumin (43 kDa), and ribonuclease A (13.7 kDa) were used as molecular weight standards for gel filtration chromatography. Majority of gC1qR/HABP1 forms trimers (C) SDS-PAGE analysis of recombinant gC1qR/HABP1. Monomers, dimers, and trimers of recombinant gC1qR/HABP1 were purified by gel permeation chromatography and analyzed by SDS-PAGE before and after reduction with βmecaptoethanol (β-ME). Molecular weights are shown in kDa.
(295 KB PDF)

Figure S2 Functional Characterization of Recombinant gC1qR/HABP1 (A) Binding of recombinant gC1qR/HABP1 to C1q. Biotinylated recombinant gC1qR/HABP1 specifically binds wells coated with human C1q. (B) Binding of recombinant gC1qR/HABP1 to HA. Biotinylated HA specifically binds to wells coated with recombinant gC1qR/HABP1. Binding to BSA-coated wells was used as control (a and B).
(206 KB PDF)

Figure S3 Binding of P. falciparum IRBCs to Purified gC1qR/HABP1 (A) Binding of IRBCs to gC1qR/HABP1 coated on plastic Petri plates. Giemsa-stained P. falciparum IGH-CR14 IRBCs are seen bound to recombinant gC1qR/HABP1 coated on plastic Petri plates. (B) Concentration-dependent binding of IRBCs to gC1qR/HABP1. Binding of P. falciparum IGH-CR14 IRBCs to gC1qR/HABP1 coated at various concentrations on plastic Petri plates. Data presented are average number of IRBCs bound per mm^2^ (± standard error) scored in duplicate spots in two independent experiments. (C) Binding of IRBCs to monomeric and trimeric gC1qR/HABP1. Binding of P. falciparum IGH-CR14 to gC1qR/HABP1 monomers and trimers purified by gel permeation chromatography is shown relative to binding to gC1qR/HABP1 containing mixed population (Mix) of monomers, dimers, and trimers. Average relative binding (± standard error) scored in duplicate spots in two independent experiments is reported.
(764 KB PDF)

Figure S4 Binding of P. falciparum IGH-CR14 to gC1qR/HABP1 in the Presence of C1q and HA (A) Binding of P. falciparum IGH-CR14 to gC1qR/HABP1 and CD36-Fc in the presence of soluble C1q is expressed as relative binding compared to binding in absence of C1q. C1q blocks binding of IGH-CR14 to gC1qR/HABP1 but does not block binding of IGH-CR14 to CD36-Fc. (B) Binding of P. falciparum IGH-CR14 to gC1qR/HABP1 in the presence of HA (1 mg/ml) is expressed as relative binding compared to binding in absence of HA. Average relative binding (± standard error) scored in duplicate spots in two independent experiments is reported.
(239 KB PDF)

Figure S5 Detection of gC1qR/HABP1 in HUVEC Cells by Western Blotting Western blotting with anti-gC1qR mouse serum (A) and anti-bcl2 rabbit serum (B). HUVEC cells were lysed by multiple cycles of freezing and thawing. Whole cell lysate (L), soluble cytoplasmic fraction (C), and insoluble membrane fraction (M) were separated by SDS-PAGE and probed for presence of gC1qR/HABP by western blotting with anti-gC1qR/HABP1 mouse serum. Recombinant gC1qR/HABP1 (rH) was used as a positive control. In a control experiment, rabbit serum raised against the mitochondrial protein, bcl-2, was used to detect any mitochondrial contamination in the membrane fraction. Anti-gC1qR mouse serum detects a protein of the expected size (32 kDa) in all three fractions, including membrane fraction. Anti-bcl2 rabbit serum only detects protein in whole cell lysate and cytosolic fractions.
(788 KB PDF)

Table S1 Detection of gC1qR/HABP1, ICAM-1, and CD36 on HUVECs, HBMECs, and PBMECs Before and After Treatment with TNF-α by Flow Cytometry (37 KB RTF)

Table S2 Detection of gC1qR/HABP1 and P-Selectin on Resting and Thrombin-Activated Platelets by Flow Cytometry (18 KB RTF)

## Abbreviations

BSA: bovine serum albumin
CHO: Chinese hamster ovary
HA: hyaluronic acid
HBMEC: human brain microvascular endothelial cell
HUVEC: human umbilical vein endothelial cell
ICAM-1: intercellular adhesion molecule-1
IRBC: infected red blood cell
PBMEC: primary brain microvascular cell
PfEMP-1: P. falciparum erythrocyte membrane protein-1
PPP: platelet-poor plasma
PRP: platelet-rich plasma
