# Supplementary figures and images for: Plasmodium falciparum Uses gC1qR/HABP1/p32 as a Receptor to Bind to Vascular Endothelium and for Platelet-Mediated Clumping
Source: PLoS Pathog. 2007 Sep 28;3(9):e130. doi: 10.1371/journal.ppat.0030130 (PMC2323294; doi:10.1371/journal.ppat.0030130)

# Supplementary Figure 1

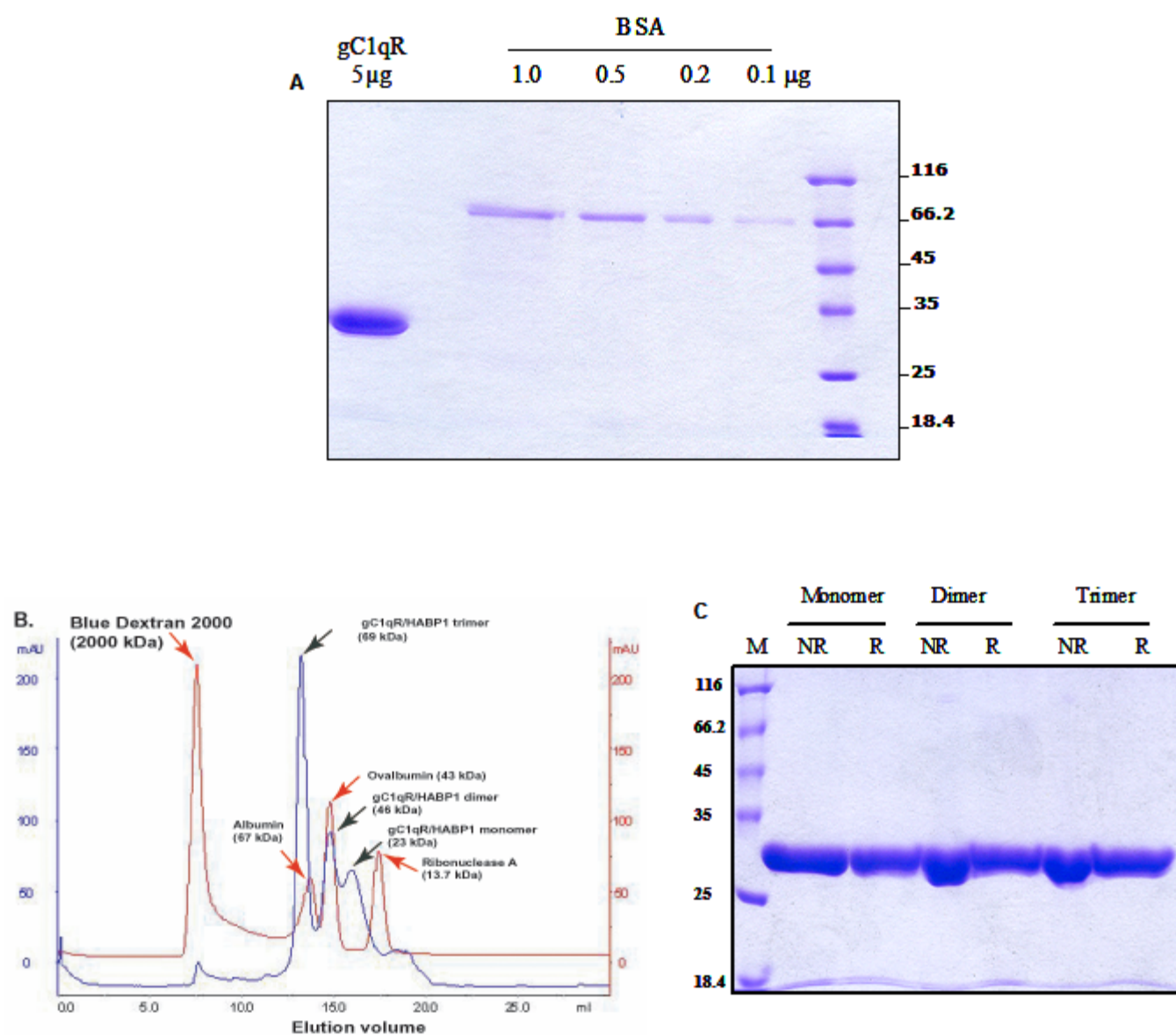

Supplement: Figure S1 — (A) Purity of recombinant gC1qR/HABP1. Purified gC1qR/HABP1 was analyzed by SDS-PAGE under reducing conditions and detected by Coomassie staining. Different amounts of BSA (0.1 μg, 0.2 μg, 0.5 μg, and 1.0 μg) were used as control. Molecular weight markers are shown in kDa. (B) Recombinant gC1qR/HABP1 forms trimers. Recombinant purified gC1qR/HABP1 was analyzed by gel permeation chromatography using Superdex 200 HR10/30 column. Theoretical molecular mass of gC1qR/HABP1 is 23 kDa. Recombinant gC1qR/HABP1 primarily migrates as a trimer with molecular weight of ∼69 kDa. Blue dextran 2000 (2,000 kDa), BSA (67 kDa), ovalbumin (43 kDa), and ribonuclease A (13.7 kDa) were used as molecular weight standards for gel filtration chromatography. Majority of gC1qR/HABP1 forms trimers (C) SDS-PAGE analysis of recombinant gC1qR/HABP1. Monomers, dimers, and trimers of recombinant gC1qR/HABP1 were purified by gel permeation chromatography and analyzed by SDS-PAGE before and after reduction with βmecaptoethanol (β-ME). Molecular weights are shown in kDa. (295 KB PDF) [file ppat.0030130.sg001.pdf]

Supplementary Figure 2

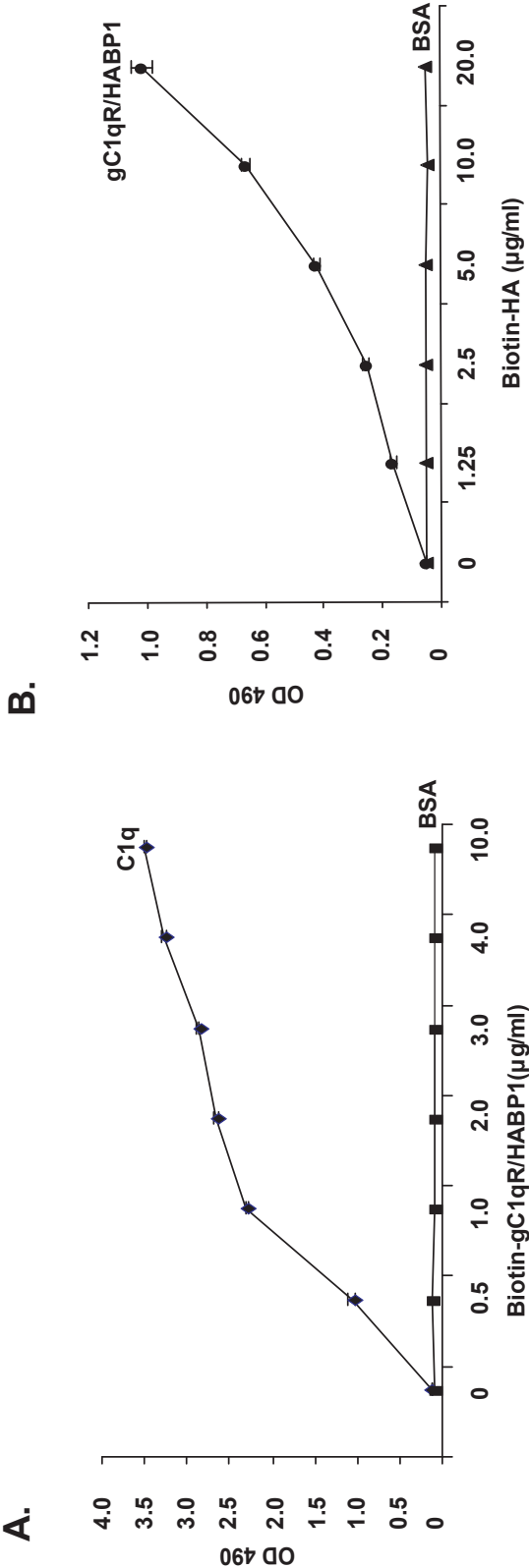

Supplement: Figure S2 — (A) Binding of recombinant gC1qR/HABP1 to C1q. Biotinylated recombinant gC1qR/HABP1 specifically binds wells coated with human C1q. (B) Binding of recombinant gC1qR/HABP1 to HA. Biotinylated HA specifically binds to wells coated with recombinant gC1qR/HABP1. Binding to BSA-coated wells was used as control (a and B). (206 KB PDF) [file ppat.0030130.sg002.pdf]

### Supplementary Figure 3

**A.**

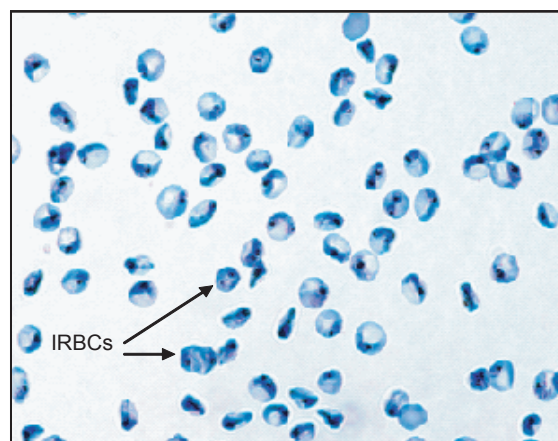

**B.**

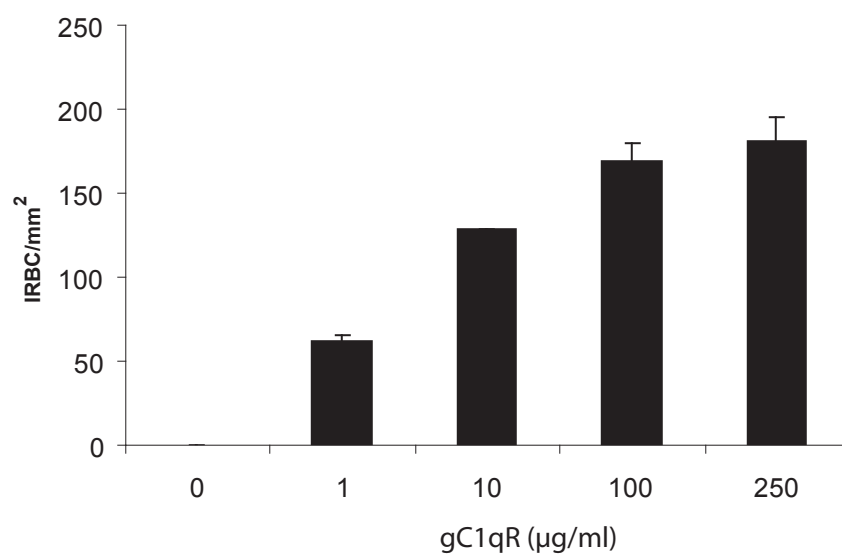

**C.**

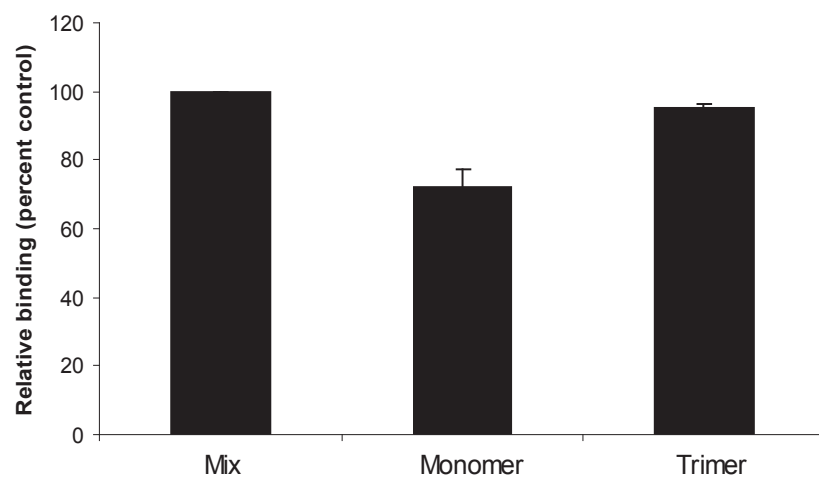

Supplement: Figure S3 — (A) Binding of IRBCs to gC1qR/HABP1 coated on plastic Petri plates. Giemsa-stained P. falciparum IGH-CR14 IRBCs are seen bound to recombinant gC1qR/HABP1 coated on plastic Petri plates. (B) Concentration-dependent binding of IRBCs to gC1qR/HABP1. Binding of P. falciparum IGH-CR14 IRBCs to gC1qR/HABP1 coated at various concentrations on plastic Petri plates. Data presented are average number of IRBCs bound per mm2 (± standard error) scored in duplicate spots in two independent experiments. (C) Binding of IRBCs to monomeric and trimeric gC1qR/HABP1. Binding of P. falciparum IGH-CR14 to gC1qR/HABP1 monomers and trimers purified by gel permeation chromatography is shown relative to binding to gC1qR/HABP1 containing mixed population (Mix) of monomers, dimers, and trimers. Average relative binding (± standard error) scored in duplicate spots in two independent experiments is reported. (764 KB PDF) [file ppat.0030130.sg003.pdf]

Supplementary Figure 4

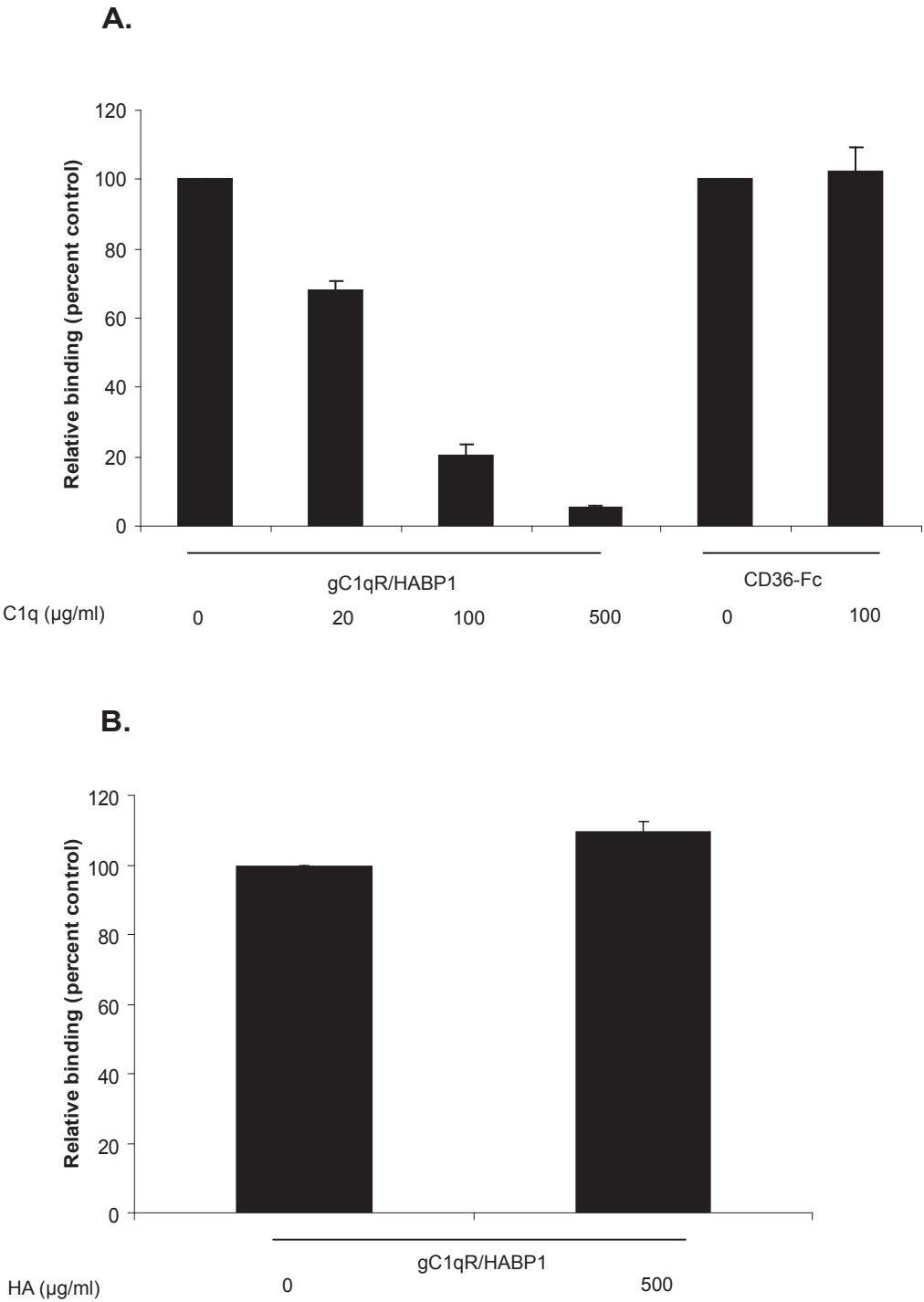

Supplement: Figure S4 — (A) Binding of P. falciparum IGH-CR14 to gC1qR/HABP1 and CD36-Fc in the presence of soluble C1q is expressed as relative binding compared to binding in absence of C1q. C1q blocks binding of IGH-CR14 to gC1qR/HABP1 but does not block binding of IGH-CR14 to CD36-Fc. (B) Binding of P. falciparum IGH-CR14 to gC1qR/HABP1 in the presence of HA (1 mg/ml) is expressed as relative binding compared to binding in absence of HA. Average relative binding (± standard error) scored in duplicate spots in two independent experiments is reported. (239 KB PDF) [file ppat.0030130.sg004.pdf]

Supplementary Figure 5

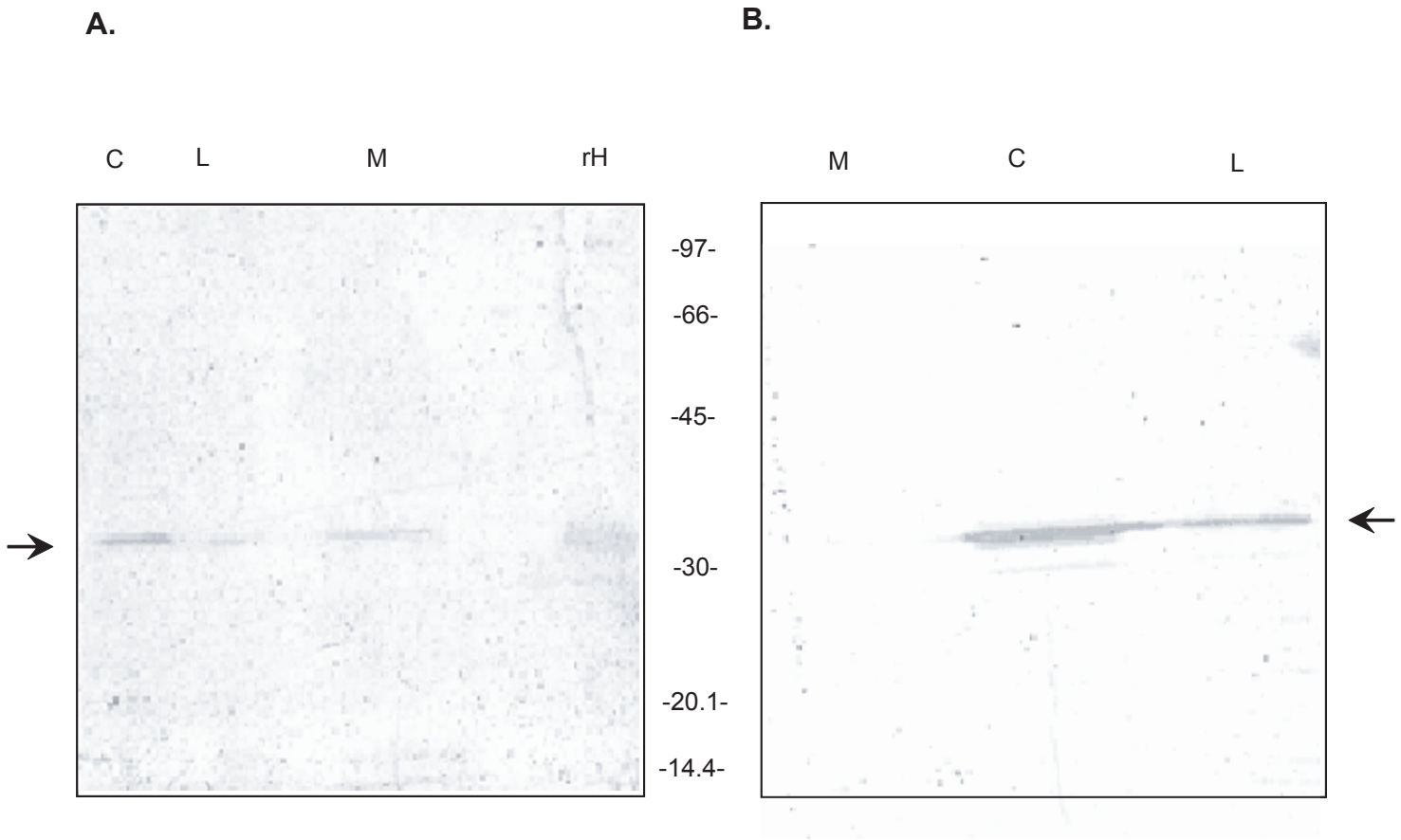

Supplement: Figure S5 — Western blotting with anti-gC1qR mouse serum (A) and anti-bcl2 rabbit serum (B). HUVEC cells were lysed by multiple cycles of freezing and thawing. Whole cell lysate (L), soluble cytoplasmic fraction (C), and insoluble membrane fraction (M) were separated by SDS-PAGE and probed for presence of gC1qR/HABP by western blotting with anti-gC1qR/HABP1 mouse serum. Recombinant gC1qR/HABP1 (rH) was used as a positive control. In a control experiment, rabbit serum raised against the mitochondrial protein, bcl-2, was used to detect any mitochondrial contamination in the membrane fraction. Anti-gC1qR mouse serum detects a protein of the expected size (32 kDa) in all three fractions, including membrane fraction. Anti-bcl2 rabbit serum only detects protein in whole cell lysate and cytosolic fractions. (788 KB PDF) [file ppat.0030130.sg005.pdf]
