# Supplementary material for: Plasmodium falciparum Uses gC1qR/HABP1/p32 as a Receptor to Bind to Vascular Endothelium and for Platelet-Mediated Clumping
Source: PLoS Pathog. 2007 Sep 28;3(9):e130. doi: 10.1371/journal.ppat.0030130 (PMC2323294; doi:10.1371/journal.ppat.0030130)
Supplement: Table S1 — (37 KB RTF) [file ppat.0030130.st001.rtf]

Supplementary Table 1. Detection of gC1qR/HABP1, ICAM1 and CD36 on HUVEC, HBMEC and PBMEC before and after treatment with TNF-a by flow cytometry.


Cell Line	
Relative Mean Fluorescence Intensity (Avg. + SD)
	
	
-TNFa
	
+TNFa	
-TNFa	
+TNFa	
-TNFa	
+TNFa	
	
PISa
	
anti-gC1qR/HABP1b
	
PIS	
anti-gC1qR/HABP1	
IgGc	
anti-ICAM1d	
IgG	
anti-ICAM1	
IgMe	
anti-CD36f	
IgM	
anti-CD36	

HUVEC	
100
	
183 + 30	
100	
186 + 29	
100 	
230 + 100	
100	
1354 + 107	
100	
94 + 6	
100	
77 + 24	

HBMEC
	
100	
146 + 9	
100	
141 + 13	
100	
206 + 28	
100	
466 + 99	
100	
73 + 17	
100	
74 + 14	
PBMEC
	100	133 + 7	100	138 + 7	100	121 + 4	100	352 + 95	100	79 + 24	100	71 + 5	
apre-immune mouse serum
banti-gC1qR/HABP1 mouse serum
ccontrol IgG
danti-ICAM1 monoclonal IgG antibody 15.2
econtrol IgM
fanti-CD36 monoclonal IgM antibody SfM.
 
