# Supplementary material for: Plasmodium falciparum Uses gC1qR/HABP1/p32 as a Receptor to Bind to Vascular Endothelium and for Platelet-Mediated Clumping
Source: PLoS Pathog. 2007 Sep 28;3(9):e130. doi: 10.1371/journal.ppat.0030130 (PMC2323294; doi:10.1371/journal.ppat.0030130)
Supplement: Table S2 — (18 KB RTF) [file ppat.0030130.st002.rtf]

Supplementary Table 2. Detection of gC1qR/HABP1 and P-selectin on resting and thrombin activated platelets by flow cytometry.

Relative Mean Fluorescence Intensity (Avg. + SD)
	

Resting Platelets
	
Activated Platelets	

PISa
	
anti-
gC1qR/HABP1b
	
Control
IgGc	
anti-
P selectind
	
PISa	
anti-
gC1qR/HABP1b	
Control
IgGc	
anti-
P selectind	

100
	
165 + 1	
100	
106 + 2	
100	
222 + 2	
100	
449 + 22	
apre-immune mouse serum; 
banti-gC1qR/HABP1 mouse serum
ccontrol IgG
danti-P selectin monoclonal IgG antibody CTB201
